# Glycan Elongation Beyond the Mucin Associated Tn Antigen Protects Tumor Cells from Immune-Mediated Killing

**DOI:** 10.1371/journal.pone.0072413

**Published:** 2013-09-06

**Authors:** Caroline B. Madsen, Kirstine Lavrsen, Catharina Steentoft, Malene B. Vester-Christensen, Henrik Clausen, Hans H. Wandall, Anders Elm Pedersen

**Affiliations:** 1 Copenhagen Center for Glycomics, Department of Cellular and Molecular Medicine, Faculty of Health and Medical Sciences, University of Copenhagen, Copenhagen, Denmark; 2 Department of International Health, Immunology and Microbiology, Faculty of Health and Medical Sciences, University of Copenhagen, Copenhagen, Denmark; King's College London, United Kingdom

## Abstract

Membrane bound mucins are up-regulated and aberrantly glycosylated during malignant transformation in many cancer cells. This results in a negatively charged glycoprotein coat which may protect cancer cells from immune surveillance. However, only limited data have so far demonstrated the critical steps in glycan elongation that make aberrantly glycosylated mucins affect the interaction between cancer cells and cytotoxic effector cells of the immune system. Tn (GalNAc-Ser/Thr), STn (NeuAcα2-6GalNAc-Ser/Thr), T (Galβ1–3GalNAc-Ser/Thr), and ST (NeuAcα2-6Galβ1–3GalNAc-Ser/Thr) antigens are recognized as cancer associated truncated glycans, and are expressed in many adenocarcinomas, e.g. breast- and pancreatic cancer cells. To investigate the role of the cancer associated glycan truncations in immune-mediated killing we created glyco-engineered breast- and pancreatic cancer cells expressing only the shortest possible mucin-like glycans (Tn and STn). Glyco-engineering was performed by zinc finger nuclease (ZFN) knockout (KO) of the Core 1 enzyme chaperone COSMC, thereby preventing glycan elongation beyond the initial GalNAc residue in O-linked glycans. We find that COSMC KO in the breast and pancreatic cancer cell lines T47D and Capan-1 increases sensitivity to both NK cell mediated antibody-dependent cellular-cytotoxicity (ADCC) and cytotoxic T lymphocyte (CTL)-mediated killing. In addition, we investigated the association between total cell surface expression of MUC1/MUC16 and NK or CTL mediated killing, and observed an inverse correlation between MUC16/MUC1 expression and the sensitivity to ADCC and CTL-mediated killing. Together, these data suggest that up-regulation of membrane bound mucins protects cells from immune mediated killing, and that particular glycosylation steps, as demonstrated for glycan elongation beyond Tn and STn, can be important for fine tuning of the immune escape mechanisms in cancer cells.

## Introduction

During malignant transformation, genetic mutations in cancer cells result in uncontrolled tumor growth, ability to metastasize, and resistance to apoptosis [1]–[3]. Concomitantly, the molecular changes may lead to induction of novel tumor associated antigens. This, together with the increasing tissue damage during tumor growth, can initiate recruitment of leukocytes into the tumor microenvironment. These infiltrating immune cells originate from both the myeloid (monocytes, dendritic cells and macrophages) [4] and the lymphoid (B cells, natural killer (NK) cells, CD4+ and CD8+ T cells) lineages [5]–[7]. The presence of such tumor infiltrating immune cells is associated with an improved prognosis in many cancer forms [8]–[11]. However, several reports indicate that the infiltrating cells such as NKs and cytotoxic T lymphocytes (CTLs) may not be able to fully eliminate established tumors [12]. Such immune escape is believed to involve mechanisms such as low immunogenicity of the tumor antigens, active immune suppression induced by regulatory T cells and myeloid derived suppressor cells (MDSCs), as well as physical barriers, e.g. encapsulation of tumors and changes in expression of membrane bound mucins [13], [14].

In the majority of adenocarcinomas, over-expression of mucin proteins, as well as alterations in mucin-type O-linked glycosylation, are observed [15]. Most mucins are secreted onto mucosal surfaces, where they function as a protective glycocalyx; while other mucins are membrane-bound due to the presence of a hydrophobic membrane-spanning domain that supports retention in the plasma membrane [15]. In particular, over-expression of the aberrantly glycosylated membrane bound mucins MUC1 and MUC16 is associated with several types of cancer [15], [16]. The extracellular domain of MUC1 consists of a variable number of 20–120 tandem repeats, each containing 20 amino acids with 5 potential O-glycosylation sites [17]. MUC16 is one of the largest mucins, with a MW ranging between 2500–5000 kDa, and contains a large and highly glycosylated tandem repeat area with over 60 repeats of a 156 amino acid sequence [18]. MUC1 and MUC16 are both glycosylated with long branched O-linked glycan structures, such as Core 2 (Galβ1-3(GlcNAcβ1-6)GalNAc) and Core 3 (GlcNAcβ1-3GalNAc), in non-malignant cells [19], [20]. In malignant cells, changes in topology, function, and expression of individual glycosyltransferases and chaperones cause loss of these elongated glycans and lead to up-regulation of short truncated glycans [19]. This results in the expression of aberrantly glycosylated mucins on the surface of cancer cells which represent cancer related antigenic neoepitopes such as Tn (GalNAc-Ser/Thr), STn (NeuAcα2-6GalNAc-Ser/Thr), and T (Galβ1-3GalNAc-Ser/Thr) [21]. It has been shown that the small tumor associated glycoepitope Tn on a MUC1 backbone is recognized by immune cells and elicits anti-tumor-antibodies [22], [23]. On the other hand, it is generally believed that high expression of aberrantly glycosylated mucins can protect cells from external assault, such as cell-mediated cytotoxicity from immune cells [15]. Although direct proof of these mechanisms is limited, it has been demonstrated that MUC16 expressed by ovarian cancer cells inhibits the formation of the immunological synapse between the tumor and NK cells [24]. In addition, core 2 O-glycosylated MUC1 carrying poly-N-acetyllactoseamine extensions impairs NK receptor interaction with tumor cells and protects bladder tumor cells from NK-mediated attack [25]. However, the exact steps in glycan elongation that are critical for protection vs. sensitivity to immune mediated killing have not been thoroughly investigated, but since aberrant glycosylation may in particular change the charge distribution, conformational dynamics, and volume of space occupied by mucins, it is anticipated to have a major influence on cellular interactions such as contact with effector cells like NK cells and CTLs [26].

In addition to the direct interaction with NK cells, cancer cell contact with monocytes in the tumor microenvironment may regulate the induction of antibody-dependent cellular-cytotoxicity (ADCC). In particular, it has recently been shown that monocytes may remove opsonizing antibodies and thus inhibit NK mediated ADCC [27], [28]. This process, described as monocyte mediated shaving, has been demonstrated for several therapeutic antibodies (Abs) including Rituximab, Trastuzumab and Cetuximab (Erbitux®) targeting CD20, human epidermal growth factor receptor 2 (HER2) and epidermal growth factor (EGF)-receptor, respectively [27]–[29]. In this respect, it is also not known how aberrant glycosylation may interfere with the negative cellular interaction between cancer cells and monocytes.

In order to examine the influence of the core 1 cancer associated glycans (T and ST) on immune-mediated killing, we have examined and compared CTL-mediated cytotoxicity and Erbitux®-mediated ADCC between cancer cells expressing the core-1 glycan structures, and cells only expressing the short cancer associated glycan structures Tn and STn. Cells lacking core 1 glycans were generated using zinc finger nuclease (ZFN) technology to eliminate the chaperone COSMC, which is essential for the function of the Core 1 synthase that elongates glycans beyond the initial Tn structure. In addition, we investigated the correlation between MUC1 and MUC16 expression and the sensitivity to ADCC and CTL-mediated killing. Finally, the influence of glycophenotype on the ability of monocytes to perform shaving of Erbitux® bound EGF-R was also investigated.

## Materials and Methods

### Ethics statement

Buffycoats were obtained from anonymous healthy blood donors. Written informed consent was obtained from the blood donors at the department of clinical immunology in Copenhagen and used without the possibility to identify case specific information. Research use for these buffycoats was approved by the ethical committee, Copenhagen county.

### Cell lines, antibodies and peptides

**Figure 1:**
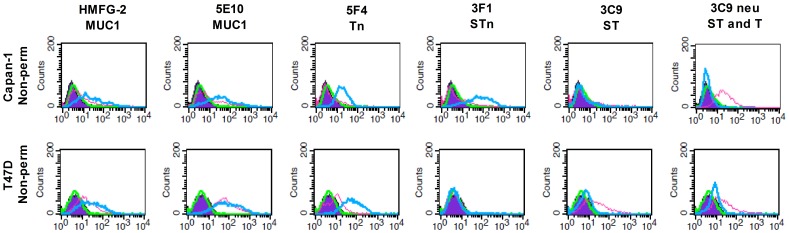
Expression profile of Capan-1 and T47D WT and COSMC KO cells. Surface/non-permeabilized (Non-perm) glycoepitope and MUC1 expression of Capan-1 and T47D WT (pink) and COSMC KO (blue) cell lines as quantified by flow cytometry using HMFG-2 (MUC1), 5E10 (MUC1), 5F4 (Tn), 3F1 (STn) and 3C9 (T) Abs. In the last row, cells have been treated with neuraminidase (neu) prior to staining, thereby removing the sialic acid from ST and allowing 3C9 to stain both the existing T structure and the former ST structures after the sialic acid removal. Isotype control for WT (purple) and KO (green) cells was used as background control.

**Figure 2:**
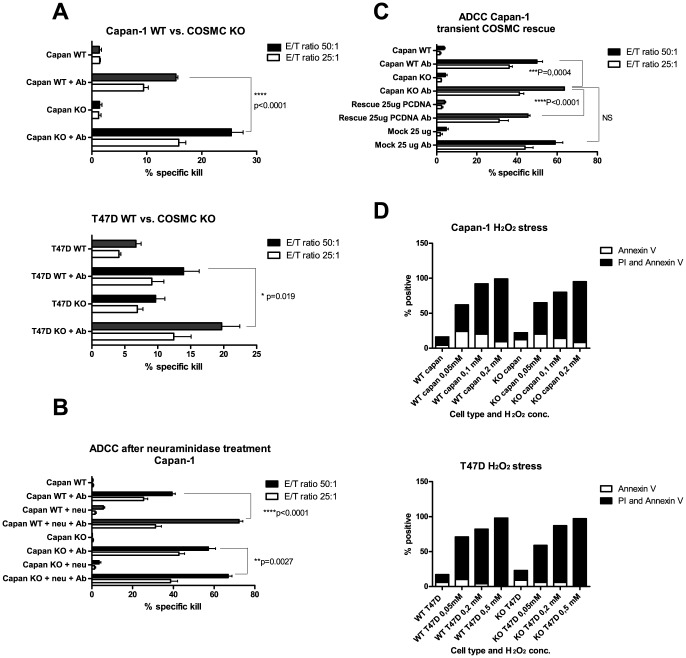
COSMC KO renders Capan-1 and T47D cells more susceptible to ADCC. A)–C) WT and COSMC KO target cells were labeled with chromium and co-cultured with PBMC effector cells in an E/T ratio of 50∶1 (black) and 25∶1 (white) either with or without Ab (Erbitux®). After 4 hrs the chromium release into the media was measured and the % specific killing was calculated. The bars show mean +/− SD from quadruplicates. Student's two-tailed unpaired t test was applied to test for significance, stars indicate level of significance. Experiments performed with two to six different PBMC donors. Data from one representative donor is shown. Experiments where cells were treated with +/− neuraminidase to remove sialic acid before ADCC is included in B) and COSMC KO cells transiently rescued with COSMC pcDNA3 transfection as well as mock transfected control are included in C). D) Capan-1 and T47D were treated with hydrogen peroxide for 20 hrs (0–0.2 mM range for Capan-1 and 0–0.5 mM range for T47D) prior to apoptosis staining using Annexin V and PI. Annexin V positive cells are considered early apoptotic whereas double positive cells are considered late apoptotic. The % positive is defined by the percent of cells above the MFI of an unstained background control in a flow cytometric analysis. One representative data set is shown out of two to three individual experiments performed.

Capan-1 cells (a kind gift from Tony Hollingsworth originally from ATCC #HTB-79) were cultured in RPMI 1640 (supplemented with 15% FBS and L-glutamine) and T47D cells (a kind gift from Tony Hollingsworth, originally published by Keydar et al. [30]) were cultured in DMEM1965 (supplemented with 10% FBS and L-glutamine). Cells were split 3 times a week after treatment with trypsin (TripLE Express from Gibco, 12605-028) and kept at maximum 95% confluence. T47D WT and KO cells were tested positive for mycoplasma but since we saw no effect on general expression patterns or apoptosis sensitivity (Figure 1 and 2) we decided that it was feasible to use the cell line in spite of the infection. Erbitux® (anti-EGF-R Ab) 5 mg/ml was a kind gift from the pharmacy at Herlev University Hospital. Glycan epitope/mucin specific Abs 5E10 (MUC1) [31], 3C9 (T) [32], 5F4 (Tn) [33], M11 (MUC16) (Dako, M3520), HMFG-2 (MUC1) [34], together with Concanavalin A (ConA)-Biotin (high mannose), Phaseolus vulgaris Leucoagglutinin (PHA-L)-Biotin (beta1,6GlcNAc-branched N-glycans), and Maackia amurensis Lectin I (MAL-1)-Biotin (sialic acid in the α2,3 linkage) were used to characterize WT and COSMC KO cell lines. In addition, FITC labeled anti-human HLA-A2 (BD pharmingen, cat # 551285), secondary rabbit anti-mouse Ig FITC (Dako (F0261)) and Streptavidin AF488 (Invitrogen, S32354) were used. Mouse IgG1 Ab (Dako (x0931)) was used as negative control. The influenza peptide GILGFVFTL was used as a model antigen in the CD8+ T cell cytotoxicity assay.

### Zink finger nuclease transfection

Stable zinc finger knockout (KO) cell lines were created as described by Steentoft et al. [35]. Briefly, cells were transfected with the COSMC ZFN custom produced by Sigma, with the following binding site and cutting site indicated in parentheses: 5′-CCCAACCAGGTAGT(AGAAGGCT)GTTGTTCAGATATGGCTGTT-3′, and selected through dilution screening using immune fluorescence. Rescue experiments of the KO phenotype were performed on cells with transient expression using pcDNA3-COSMC, using pcDNA3 alone as transfection control. ST expression was restored in 25–50% of the cells transiently transfected with the pcDNA3-COSMC construct.

### pcDNA3-COSMC construction

A full length FLAG tagged COSMC construct was generated using primers; COSMC1 (5′-GCGGAATTCAAAATGCTTTCTGAAAGCAGCTCC-3′)/COSMC3 (5′-GCGCGCGGCCGCCTACTTATCGTCGTCATCCTTGTAGTCATTGTCAGAACCATTTGGAGGTAAG-3′) flanked by EcoRI and NotI restriction sites respectively. In addition COSMC3 encoded a FLAG epitope tag. IMAGE clone IRAUp969H0363D was used as template together with Pfu-UltraII polymerase (Agilent Technologies, CA, USA) under supplier recommended amplification conditions. Amplicon was inserted into the EcoRI/NotI site of pcDNATM3.1/hygro (Life Technologies, UK) and sequence validated. As mock transfection control pcDNATM3.1/hygro (Life Technologies, UK) was used without insert.

### Monocyte purification and CTL expansion

Monocytes were purified from human peripheral blood mononuclear cells (PBMC), isolated from blood donor buffy coats obtained from The Department of Clinical Immunology at Copenhagen University Hospital. The buffy coat was separated on Lymphoprep (Fresenius Kabi, Norway) and the PBMC layer was salvaged and washed before the monocytes were purified by MACS CD14 microbeads (Miltenyi Biotec, Cat # 130-050-201) following the manufacture's recommendations. HLA-A2 positive blood donor PBMCs were stimulated with influenza peptide GILGFVFTL for 24 hrs *in vitro* (with 2% human AB serum (Valley Biomedical Inc. HS1017) to induce cytotoxic T cell clones. Following peptide stimulation, the cells were washed and set up in 96 well plates with 50 U/ml recombinant IL-2 for an additional 7 days. The expanded cells enriched for influenza peptide specific CD8+ T cells were used in the chromium release assay with WT or COSMC KO cells as target cells.

### ADCC and CTL-mediated cytotoxicity

Capan-1 and T47D cells were used as target cells in the ADCC assay after trypsinizing with TripLE Express (Gibco. 12605-028) at 90% confluence, and were transferred into 6 well plates with 0.8*10^6^ cells/well with the addition of 2.5 ug/ml of Erbitux® or irrelevant human IgG and 4*10^6^ purified monocytes when indicated (in shaving experiments). Effector PBMCs were purified from buffy coats and set up in RPMI 1640 with 10% human AB serum (Valley Biomedical Inc. HS1017) as 3*10^6^ cells/ml in 6 well plates. The cells were left in the incubator for 18–20 hrs. The wells were then washed to remove monocytes (in shaving experiments) and excess Erbitux® and the cells were trypsinized. For CTL cytotoxicity assays the WT and COSMC KO cancer cells were pulsed for 4 hrs with the GILGFVFTL influenza peptide. Next, the cancer cells were labeled with radioactive chromium and used as target cells in a standard 4 hr chromium release assay. The PBMCs from day one were used as effector cells in the ADCC assay and the *in vitro* expanded influenza peptide specific CD8+ T cells were used in the CTL cytotoxicity assay.

### Flow cytometry

Cells were detached with TripLE Express (Gibco. 12605-028) and washed once in media containing 10% FBS. Cells were washed 1–2 times in cold PBS and for secondary labeling they were stained with primary Ab for 1 hr and secondary Ab for 40 min in the dark. Prior to labeling, the non-permeabilized T47D cells were fixed in 4% paraformaldehyde. For intracellular staining the BD CytofixCytoperm kit (Cat # 554715) was used following the manufacture's recommendations. Where mentioned, cells were treated with 0.1 U/ml neuraminidase at 37°C for 1 hr prior to staining. For direct labeling, the cells were stained for 20 min on ice in the dark. After washing, the cells were analyzed on a BD Facs Calibur using CellQuest Pro software. For flow cytometry based ADCC and CTL experiments the Capan-1 cells were labeled with carboxyfluorescein succinimidyl ester (CFSE) (Sigma) one day prior to set up with PBMCs or CTLs. After 4 hr incubation, the cells were stained with the appropriate anti-mucin Ab and propidium iodide prior to the flow cytometric analysis.

### H_2_O_2_ stress assay

Capan-1 and T47D cells were set up in 6 well plates with 0.8*10^6^ cells per well and a gradient of hydrogen peroxide was added from 0–0.5 mM. After 20 hrs in a humidified incubator at 37°C 5% CO_2_, the cells were removed from the wells with TripLE Express (Gibco, 12605-028), washed and stained with the Annexin V-FITC apoptosis detection kit I (BD pharmingen, cat number: 556547) following the manufacturer's recommendations. The cells were analyzed on a BD Facs Calibur using Cell Quest Pro software.

### Proliferation assays

Capan-1 cells were set up in titrated numbers from 625–40,000 cells per well in a 96 well plate in RPMI 1640 (supplemented with 15% FBS and L-glutamine) in quadruplicates. 20 µl 0,125 µCi/ml of 3H-thymidine (Perkin Elmer, NET027X005MC) was added per well and the plate was incubated at 37°C for 18 H in a humidified incubator. The cells were harvested onto filter plates, scintillation fluid (Microscint O, Perkin Elmer) was added and the counts quantified using the TopCount (Packard BioScience). T47D cells were set up in titration in triplicates from 1,000–10,000 cells/well in a 96 well plate in DMEM 1965 (supplemented with 10% FBS and L-glutamine), left to proliferate for 48–72 hrs, washed in PBS, frozen at −80°C and quantified with CyQuant® (Invitrogen, MP 07026) following the manufacturer's recommendations.

### Statistical analysis

For individual experiments the data are presented as mean +/− SD and the students unpaired two tailed t test is applied to test for significance while pooled data from several donors are depicted as mean +/− SEM and a paired students two tailed t test is applied to test for significance.

## Results

### Characterization of WT and KO cancer cells

COSMC KO cells were generated from Capan-1 and T47D cells by ZFN strategies and single cell cloning [35]. COSMC KO cells only express the Tn/STn antigen, while the WT cancer cells primarily express sialylated T structures (Figure 1A and Figure S1). WT and COSMC KO cells express similar levels of MUC1 in both cell lines, although the level is slightly elevated in Capan-1 COSMC KO cells (Figure 1A and Figure S1). In contrast, MUC16 is only expressed in Capan-1 but with similar expression between COSMC KO and WT cells (Figure S2)). Like the MUC1 expression, the level of MUC16 is also very heterogeneous in both WT and COSMC KO cells (Figure S2). HLA-A2 was only expressed in Capan-1, but at comparable levels in WT and COSMC KO cells, albeit the WT had a slightly higher expression. The EGF-receptor is expressed at similar levels in WT and COSMC KO cells of both Capan-1 and T47D (Figure S2). Staining of the Capan-1 cells with the lectins, ConA, MAL-1 and PHA-L that primarily recognize different parts of N-linked glycan branches showed similar staining between WT and COSMC KO cells, suggesting that N-glycan expression is not affected by COSMC elimination (Figure S4).

Finally, an evaluation of the proliferative potential of WT and COSMC KO Capan-1 and T47D cells revealed equal proliferation of WT and COSMC KO cells (Figure S3).

### COSMC KO cancer cells are more susceptible to ADCC than their WT counter parts

Using the therapeutic anti-EGF-R Ab Erbitux® we investigated the ability of donor PBMCs to kill the WT and COSMC KO cells in an ADCC assay. Both the Capan-1 and the T47D COSMC KO cells were more susceptible to ADCC than their WT counter parts, with an increase in the specific killing of KO versus WT cells of 6% (p = 0.019) for T47D and 10% (p<0.0001) for Capan-1 with the donors used in Figure 2A. In Table S1, cumulative data from all donors are presented and support this finding. To test if the increased ADCC mediated killing of COSMC KO cells was due to a reduction in sialylated O-linked glycans, we reduced surface sialic acid by neuraminidase treatment and monitored ADCC of the WT and the COSMC KO Capan-1 cells. After treatment with neuraminidase both WT and COSMC KO Capan-1 cells became more susceptible to ADCC (Figure 2B and Table S1). The effect was best observed in WT cells where an increase in specific killing of almost 30% (p<0.0001) was identified (Figure 2B). To confirm that the increased ADCC of the COSMC KO cells was due to the lack of ST structures we re-introduced COSMC by transient transfection in Capan-1 cells. Rescue of the ST structure, as well as a decrease in ADCC of the COSMC rescued Capan-1 cells, were demonstrated, while mock transfection with the PCDNA3 construct alone did not affect the killing (Figure 2C and Figure S5). Capan-1 and T47D cells treated with irrelevant control human IgG were not targeted by ADCC (data not shown). In addition, we compared apoptosis of WT and COSMC KO cells induced by oxidative stress. Both Capan-1 and T47D WT and COSMC KO cells were equally prone to hydrogen peroxide induced apoptosis (Figure 2D), excluding that the increased killing of the COSMC KO clones was due to a general increase in sensitivity to apoptosis.

### COSMC KO cancer cells are more susceptible to CTL-mediated killing than their WT counterparts

**Figure 3:**
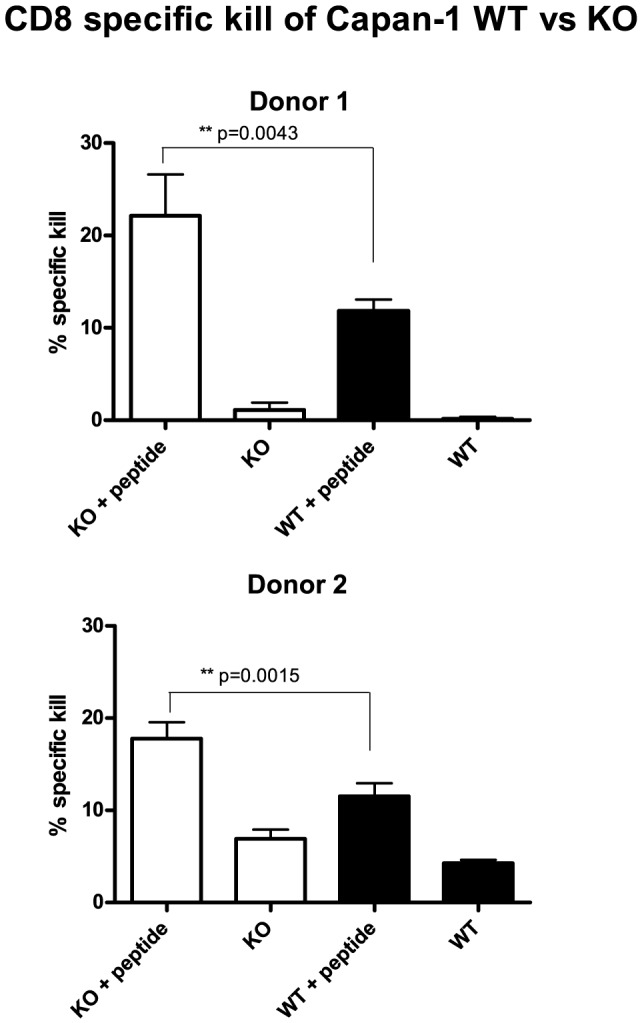
Capan-1 COSMC KO cells are more susceptible to CTL-mediated killing than Capan-1 WT. Chromium release assay with Capan-1 WT/Cosmc KO target cells either (GILGFVFTL ) influenza peptide loaded or not. Influenza peptide primed CD8+ T cells were used as effector cells in an E∶T ratio of 4∶1. The bars show mean +/− SD of the calculated specific killing from quadruplicates. Student's two-tailed t test was applied to test for significance; stars indicate level of significance. Results from two out of three donors are shown.

To evaluate the influence of COSMC KO on susceptibility to CTL-mediated killing, PBMCs were obtained from HLA-A2 positive donors and re-expanded with GILGFVFTL influenza peptide stimulation *in vitro* for 7 days. Subsequently, the expanded PBMCs were used as effector cells in a chromium-release assay where the Capan-1 cells were loaded with the GILGFVFTL peptide. COSMC KO cells were more susceptible to CTL kill than WT cells, resulting in a 6–10% (maximum p value of 0.0043) increase in specific killing (Figure 3).

### Susceptibility to ADCC and CTL is associated with expression levels of MUC16 and MUC1

**Figure 4:**
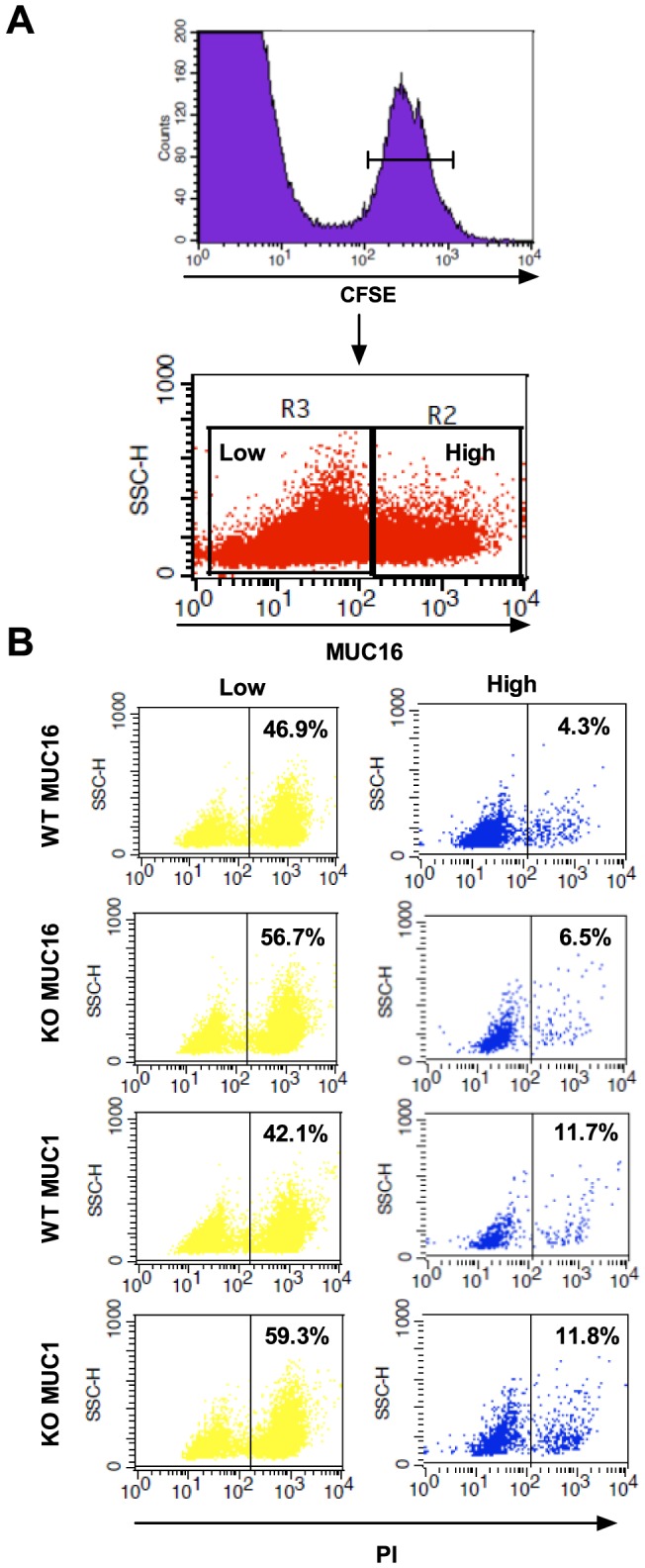
MUC1 and 16 expression is inversely correlated with sensitivity to ADCC-mediated killing. CFSE labeled Capan-1 cells stained for MUC1 or MUC16 expression and propidium iodide (PI) uptake after 4 hrs co-culture with Erbitux® and PBMCs as analyzed by flow cytometry. A) Gating strategy on CFSE positive Capan-1 cells and from that population the selection of high and low MUC16 expressing cells. The gating strategy is the same for the MUC1 stained cells. B) The % PI positive cells are quantified in the MUC1/16 high and low expressor populations within the CFSE positive Capan-1 WT or COSMC KO cell population. Experiments performed with six different PBMC donors. Data from one representative donor is shown.

**Figure 5:**
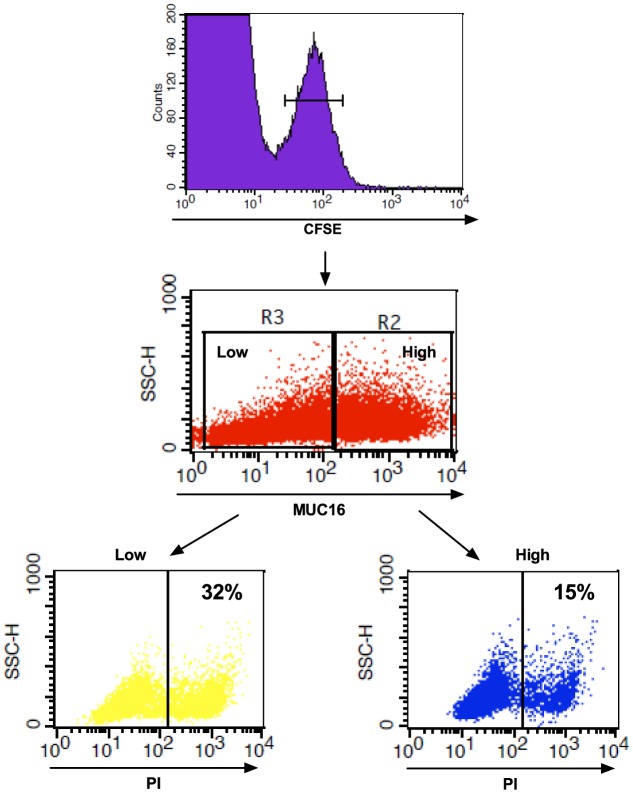
MUC16 expression is inversely correlated with sensitivity to CTL-mediated killing. Influenza peptide pulsed CFSE labeled Capan-1 cells stained for MUC16 expression and propidium iodide (PI) uptake after 4 hrs co-culture with influenza peptide specific CTLs as analyzed by flow cytometry. The % PI positive cells are quantified in the MUC16 high and low expressor populations within the CFSE positive Capan-1 WT cells. Experiments performed with four different PBMC donors. Data from one representative donor is shown.

As seen from Figure 1 the expression of MUC1 and MUC16 is heterogeneous on both the WT and the KO cancer cells. Therefore, we investigated the ADCC-mediated killing in the mucin high and low expressing target cell populations. Before initiation of the experiments, we verified that mucin expression was not correlated with the expression of either EGF-R or HLA-A2 (data not shown). Using a flow cytometry based assay, the differentiated ADCC killing of low and high MUC16 and MUC1 expressing Capan-1 cells was investigated. CFSE labeled Capan-1 WT and COSMC KO cells were co-cultured with PBMCs after Erbitux® labeling in a E∶T ratio of 50∶1. After 4 hrs incubation the induced death was measured by the PI uptake of the CFSE labeled Capan-1 cells. MUC16 and MUC1 expression was inversely correlated with sensitivity to ADCC (Figure 4 and Figure S6). Additionally, the MUC1 and MUC16 low expressing COSMC KO cells were significantly more susceptible to ADCC than their WT counterparts (Figure S6). Due to the abnormally large size of the MUC16 mucin the correlation of MUC16 expression with CTL-mediated killing was also investigated. The Capan-1 cells were loaded with influenza peptide after CFSE labeling, put into co-culture with influenza peptide specific CTLs at an E∶T ratio of 40∶1 and the PI uptake was measured as for the ADCC. It was found that MUC16 expression was inversely correlated with CTL-mediated killing as well (Figure 5 and Figure S7).

### Monocyte-mediated shaving is not affected by Cosmc KO

**Figure 6:**
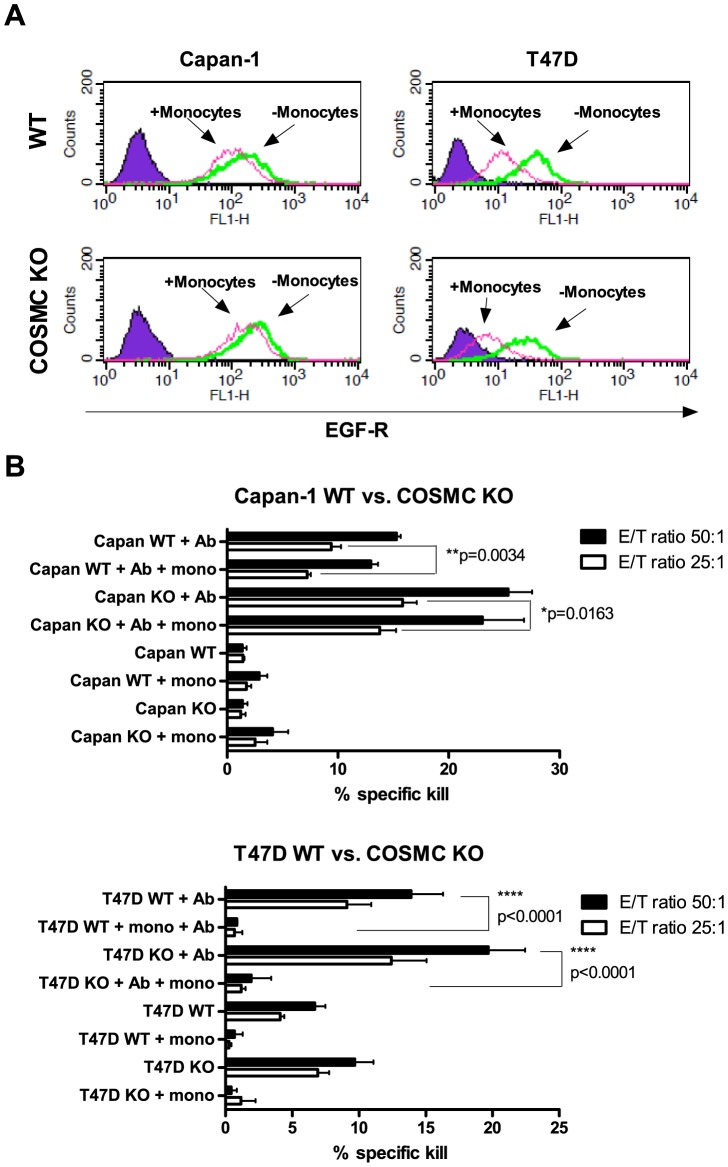
Monocyte-mediated shaving is not affected by COSMC KO. A) Histogram plots representing the fluorescent intensity of the EGF-R expression as determined by Erbitux® binding. Left panel shows Capan-1 WT and COSMC KO, right panel T47D WT and COSMC KO. Secondary Ab staining alone was used as a background control (purple). Cells treated with Erbitux® Ab were put into culture for 20 hrs either with (pink) or without (green) monocytes, and subsequently stained for remaining Erbitux® and analyzed by flow cytometry. B) ADCC on cells set up as in Figure 2 with additional groups of target cells treated with monocytes for 20 hrs after Erbitux® labeling. The bars show mean +/− SD from quadruplicates. A student's two-tailed t test was applied to test for significance, stars indicate level of significance. A representative data set is shown out of experiments performed with five to six different PBMC donors.

We next evaluated the effect of glyco-phenotype on sensitivity to monocyte-mediated shaving of opsonizing Abs. Monocytes were added to the T47D or Capan-1 culture for 20 hrs after the cells had been labeled with Erbitux® Ab. The remaining EGF-receptor-Erbitux® complexes were quantified by antibody staining followed by flow cytometry analyses. The monocytes shaved EGF-receptor-Erbitux® complexes at similar levels for Capan-1 and T47D (MFI difference of approx. 50 and 22 for WT Capan-1 and T47D respectively). There was no difference in the amount of monocyte-mediated shaving (Figure 6A) of WT and COSMC KO cells (Figure 6A) as the difference in MFI with or without monocyte treatment were ∼50 for both Capan-1 WT and COSMC KO and ∼22 for T47D WT and COSMC KO cells. To investigate a potential effect of monocyte mediated shaving on subsequent NK mediated killing, ADCC assays of the monocyte treated WT and COSMC KO cells were performed. Erbitux® opsonized cancer cells co-cultured with monocytes are less prone to ADCC than Erbitux® opsonized cancer cells alone, confirming the negative effect of shaving, but no difference was seen in the shaving effect between WT and COSMC KO cells (Figure 6B). The ADCC of T47D cells seems to be affected to a higher extent by monocyte shaving than Capan-1 cells, potentially due to the lower EGF-R expression as compared to Capan-1 cells, but again there was no difference between WT and KO cells. Serving as negative controls, no significant difference in ADCC was observed between non-opsonized WT and COSMC KO cells co-cultured with monocytes. Additionally, we examined the monocyte shaving of mucin high and low expressing cells by flow cytometry and observed no difference in the level of shaving in the MUC1/16 high and low expressing population (data not shown). Thus, monocyte-mediated shaving is not affected by the replacement of the longer ST and T structures with the shorter Tn/STn structures.

## Discussion

The classic mechanisms of tumor immune evasion include down-regulation of MHC expression, which leads to escape from CD8+ T cells [36], internalization of opsonizing Abs, recruitment of regulatory T cells and MDSCs, as well as the creation of an immune suppressive tumor microenvironment by secretion of anti-inflammatory cytokines in general [37]–[39]. Here, we demonstrate that elongated Core 1 structures of membrane bound mucins on the surface of cancer cells might also be critical in protecting tumor cells against immune-mediated attack. To our knowledge this is the first study to investigate how the short glycans (e.g. Tn and STn) and glycans of intermediate length (e.g. the Core-1 glycans T and ST) affect immune mediated killing from ADCC and CTLs. To dissect the role of the individual glycan structures, we used ZFN constructs to knock out the chaperone COSMC required for O-linked glycans beyond the initial GalNAc and compared ADCC- and CTL-mediated killing of cancer cells with and without Core 1 O-glycans. Furthermore we investigated the effect on monocyte mediated shaving of opsonizing antibodies. Lastly, the association between total expression levels of large mucins like MUC1 and MUC16 and immune mediated killing was examined.

We observed reduced ADCC and CTL-mediated killing of WT breast and pancreas cells expressing the ST and T/Core 1 structure as compared to COSMC KO cells displaying only short cancer associated glycans STn and Tn (Figure 2 and 3, Table S1). Importantly, the increase in ADCC was not caused by differences in apoptosis sensitivity or differences in proliferation capacity, and could be rescued by transient knockin of the COSMC chaperone. The rescued COSMC KO cells have a slightly lower ADCC sensitivity than the WT cells, possibly explained by an increase in ST expression compared with the WT cells and/or by the slight increase in the basal level of MUC1 expression in the KO cells. The finding that elongation of glycans can protect cancer cells from ADCC is in accordance with the demonstration that Core 2 glycans can mediate protection from immune-mediated killing of cancer cells [25]. This could suggest a biological advantage for cells expressing Core 1 or 2 structures. Nevertheless, most cancer cells predominantly express the shorter O-glycans Tn and STn as well as the Core 1 based O-glycans T and ST (reviewed in [19]). Therefore, there must be a prominent growth or survival advantage associated with the expression of these truncated Tn and STn glycans. Indeed it has been shown that expression of the STn structure increases proliferation and metastatic potential of cancer cells [19], [40], [41]. The mucin up-regulation seen in most adenocarcinomas might then serve as the main immune shielding mediator while the glyco-phenotype promotes proliferation and metastasis. Such interplay of different truncated glycans and mucins are consistent with the heterogeneous expression profile seen in many tumors.

To investigate the role of sialic acid, we evaluated ADCC of the Capan-1 cells before and after reduction of the amount of surface sialic acid by neuraminidase treatment. Based on our findings, we can conclude that sialylation protects against immune-mediated killing of cancer cells (Figure 2B and Table S1). Furthermore, neuraminidase treatment of WT cells expressing ST and COSMC KO cells expressing STn demonstrates that the protective capacity of sialic acid is greatest if present on T compared to Tn structures (Figure 2B and Table S1). It should be mentioned that, due to intra donor variations, only the data from individual experiments exhibited significant differences (Table S1). These findings are consistent with data indicating that sialic acid is a cellular label of self, which acts as an anti-recognition agent that may shield glycoproteins from receptor binding, e.g asialoglycoprotein receptor and CD44, under normal conditions [42], [43]. Sialic acid might also facilitate protection from immune mediated killing through binding to inhibitory receptors expressed on NK cells such as the Siglec receptors [44], [45]. Finally, the shielding effect of sialic acid could also be explained by the repulsive forces induced by the negative charge of sialic acid. For example, sialylation of endothelial cells in developing blood vessels has been shown to create repulsion able to initiate lumen formation [46], and polysialylation of neural cell adhesion molecules increases intermembrane repulsion [47], [48]. Taken together, these observations suggest that removal of sialic acid could also render cancer cells more prone to NK cell-mediated killing.

CTL-mediated killing and ADCC involve synapse formation between the cytotoxic cell and target cell mediated by a number of different receptor-ligand interactions [48]–[50]. For ADCC, only a few of these have been characterized, e.g. the stabilization of the Ab-CD16 interaction by ICAM-1–LFA-1 [50], [51], while the adhesion molecules and co-receptors in the formation of the immunological synapse between CTLs and target cells are well known [48]. Because most proteins involved in the synapse are glycoproteins, we cannot exclude that the lack of elongated O-linked glycans in COSMC KO cells influences the level of surface expression, function and/or interactions of proteins in the synapse. However, we did not observe any difference in the total expression of the EGF-R, HLA-A2, MHC class I polypeptide-related sequence A/B and UL16-binding protein-2 expression (Figure S2 or data not shown); and furthermore, the expression patterns of the two membrane associated mucins MUC1 and MUC16 were similar, which suggests that the total expression levels are unaltered between COSMC KO and WT cells. Additionally, many of the proteins involved in the synapse are N-glycosylated and, even though it might be possible that elimination of elongated O-glycans will alter the concentration of donor sugars in the Golgi apparatus, we see no overall alteration in either ConA, MAL-1 or PHA-L staining of the WT and KO cells, indicating that surface N-glycans were unaffected by COSMC KO (Figure S4).

Expression of the large and heavily glycosylated MUC16 mucin has previously been shown to interfere with the synapse formation between ovarian cancer cells and NK cells [24]; and the rat homologue of MUC4, the sialomucin complex, has been shown to shield cancer cells from lymphokine activated killer cells, thereby increasing their metastatic potential [52], [53]. Since the pancreatic cancer cells used in this study have high, albeit heterogeneous, MUC16 expression, in addition to heterogeneous MUC1 expression (Figure 1), we wanted to investigate the immune shielding effect of MUC1 and MUC16 in regard to ADCC. Initially we tried to establish MUC1 and MUC16 ZFN KOs of the cancer cells, but difficulties in generating the ZFN MUC1 and MUC16 KO cells prevented us from using this approach (data not shown). Therefore, we decided to use the naturally heterogeneous expression of the mucins and evaluate the sensitivity to immune mediated killing in the high and low mucin expressing populations. With this approach, we show that sensitivity to ADCC and CTL-mediated killing is inversely correlated with MUC16 expression, and that ADCC sensitivity is inversely correlated with MUC1 expression (Figure 4 and 5, Figure S6 and S7). This was observed in all individual donors as well as for the cumulative data set of all 4–5 donors. However, it remains unclear if the effect is caused by specific receptor interactions, the large size of the mucin molecules, or the up-regulated aberrant glycosylation of these mucins. Indeed, the immune shielding effect of MUC16 might be partially caused by the inhibitory effect of MUC16 binding to Siglec 9 on NK cells [54]. However, NK cell lines lacking Siglec 9 still target MUC16 low expressing cells, suggesting that other factors, e.g. mucin size, could explain the inhibitory effect of MUC16. Close proximity between NK cells and target cells is required (10–55 nm) to induce cytotoxicity [55], but mucins protrude from the cell surface by an estimated 7 nm per 28 amino acids [26], and hence may interfere with or abolish NK cell accessibility. Thus, when MUC1 expressing T cells interact with DCs, active mechanisms are needed to polarize MUC1 expression to sites opposing the DC-T cell synapse [56].

Shaving, or trogocytosis, is performed by monocytes in the presence of an Ab bound to a cellular target [27]–[29]. Accordingly, this mechanism may reduce the effect of opsonizing Abs in tumor immune responses, causing ineffectiveness of immunotherapies that take advantage of NK mediated ADCC [28], [57]. The mechanisms of monocyte mediated shaving/trogocytosis are not well established but appear to involve a receptor-mediated endocytotic process where the plasma membranes of the monocyte and cancer cell fuse at the site of FcγR-Ab binding, and the opzonizing Ab and its target are removed from the cancer cell, along with parts of the cancer cell's plasma membrane [27], [29], [58]. The possible effect of the glycophenotype on monocyte mediated shaving has not been investigated previously. Here, we tested if elongation beyond Tn could affect shaving similar to the effect observed for ADCC and CTL-mediated attack. We confirmed that Erbitux® bound to Capan-1 and T47D was shaved off by monocytes in the co-culture, but the level of shaving/trogocytosis was not affected by the glycosylation state of the cancer cell (Figure 6A). However, the observed amount of Erbitux® shaving/trogocytosis is much smaller than that normally seen for Rituximab shaving [28]. This might be caused by limited monocyte access to the cells with high MUC1/16 levels, resulting in effective shaving only of cells with low expression of these mucins. However, we did not observe any significant difference in the degree of shaving between high- and low-mucin expressing cells (data not shown), and are therefore unable to offer a thorough explanation for our findings at this point. Also, the MUC16 expressing Capan-1 cells show a slightly higher susceptibility to shaving after Erbitux® staining compared to the MUC16 neg T47D cells (MFI difference of ∼50 (Capan-1) and ∼22 (T47D)), which further supports that mucins do not have a major influence on shaving. Although, it may appear from Fig. 6B that the T47D cells are less susceptible to ADCC after monocyte shaving compared to Capan-1 cells, we believe that this is caused by the initial lower EGF-R expression on T47D cells.

In conclusion, cancer cells (Capan and T47D) carrying the short truncated cancer associated glycans (Tn and STn) are more susceptible to ADCC and CTL-mediated killing than cells carrying the longer Core 1 carbohydrate structures. Furthermore, general up-regulation of MUC1 and MUC16, independent of specific glycan expression, protects Capan-1 cancer cells from ADCC and CTL-mediated killing. In general, both mucin expression, length of carbohydrates, and degree of sialylation protects cells from immune mediated killing.

## Supporting Information

Figure S1 **Facs data of permeabilized T47D and Capan-1 cells, equivalent to non-permeabilized cells shown in** **Figure 1** **.**
(PDF)

Figure S2 **Target expression profile.** Flow cytometry staining using non-perm WT (green) and COSMC KO (pink) cells to quantify the surface expression of HLA-A2, EGF-R (Erbitux®) and MUC16 (M11). Isotype control for WT (purple) and KO (blue) was used as background control.
(PDF)

Figure S3 **Proliferation profile for Capan-1 and T47D WT and COSMC KO cells.** A) Thymidine(3H) incorporation is shown for individual cell concentrations of WT and COSMC KO Capan-1 cells after 18 H of incubation. Cpm: counts per minute. Representative of two individual experiments on two different cell passage numbers/batches. B) Fluorescence intensity in wells after CyQuant® proliferation assay of T47D WT and COSMC KO cells. Representative data set of 3 individual experiments.
(PDF)

Figure S4 **Lectin staining of WT and COSMC KO capan-1 cells.** Flow cytometric staining of WT (pink) and KO (blue) cells with ConA, PHA-L and MAL-1. Non stained (purple) and streptavidin alone (green) used as background.
(PDF)

Figure S5 **Data on additional rescue setup.** Transfections performed with different batches of PCDNA3 construct and different concentrations. Analysis as in figure 2.
(PDF)

Figure S6 **Pooled ADCC data on mucin high and low expressing cells.** Equivalent % specific kill as depicted in figure 4. Paired students t-test results in significant difference with WT MUC1 High/Low: *P = 0.02, KO MUC1 High/Low: **P = 0.0082, MUC1 Low WT/KO: **P = 0.0012, WT MUC16 High/Low: **P = 0.0092, KO MUC16 High/Low: **P = 0.0024, and MUC16 Low WT/KO: *P = 0,013.
(PDF)

Figure S7 **Pooled CD8+ T cell kill data on MUC16 high and low expressing cells.** Equivalent % specific kill as depicted in figure 5. Paired students t-test results in significant difference with P = 0,0016.
(PDF)

Table S1 **ADCC data for all donors, of which representative donors are shown in** **figure 2** **.** Stars indicate level of significance. P value for individual experiments obtained by unpaired students t test, while P value for cumulative data (last row) was obtained by paired students t test. § average % specific kill was slightly above 100%, set to 100% in analysis. N/A: not available, N/S: not significant.
(PDF)
