# Correction: Glycan Elongation Beyond the Mucin Associated Tn Antigen Protects Tumor Cells from Immune-Mediated Killing

**DOI:** 10.1371/annotation/81b54c99-e47f-4128-8aeb-b6b3e8c02562

**Published:** 2013-10-01

**Authors:** Caroline B. Madsen, Kirstine Lavrsen, Catharina Steentoft, Malene B. Vester-Christensen, Henrik Clausen, Hans H. Wandall, Anders Elm Pedersen

As a result of problems in the production process, there were errors in the version of Figure 2 that appears in the PDF version of the article. The version that appears in the online version of the article is correct. 

