# Supplementary figures and images for: Glycan Elongation Beyond the Mucin Associated Tn Antigen Protects Tumor Cells from Immune-Mediated Killing
Source: PLoS One. 2013 Sep 6;8(9):e72413. doi: 10.1371/journal.pone.0072413 (PMC3765166; doi:10.1371/journal.pone.0072413)

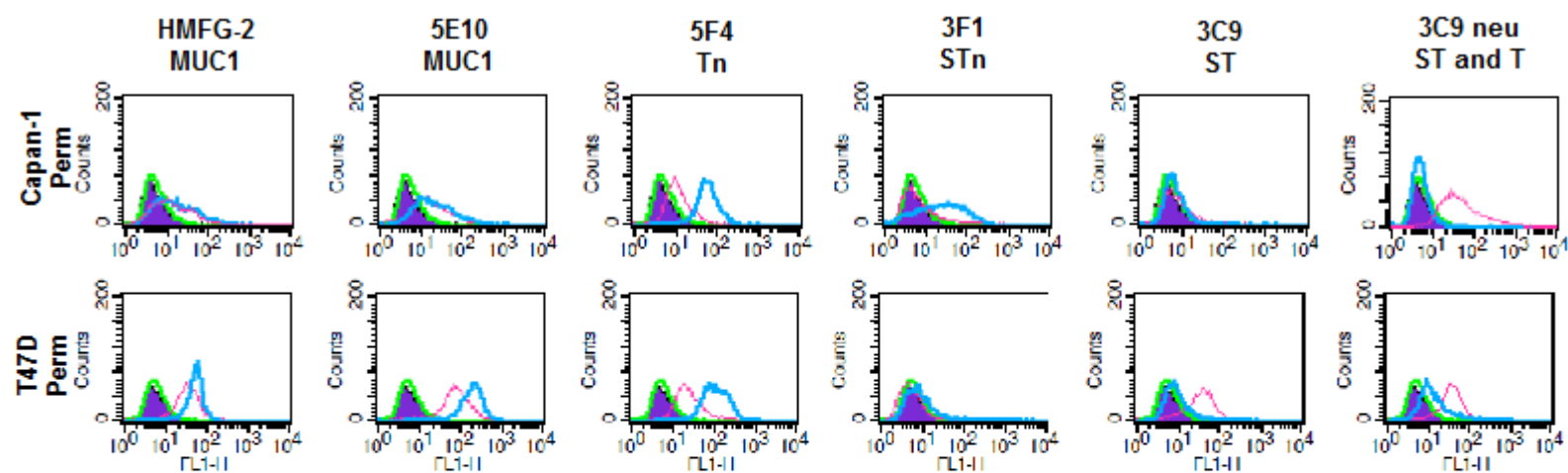

Supplement: Figure S1 — Facs data of permeabilized T47D and Capan-1 cells, equivalent to non-permeabilized cells shown in Figure 1 . (PDF) [file pone.0072413.s001.pdf]

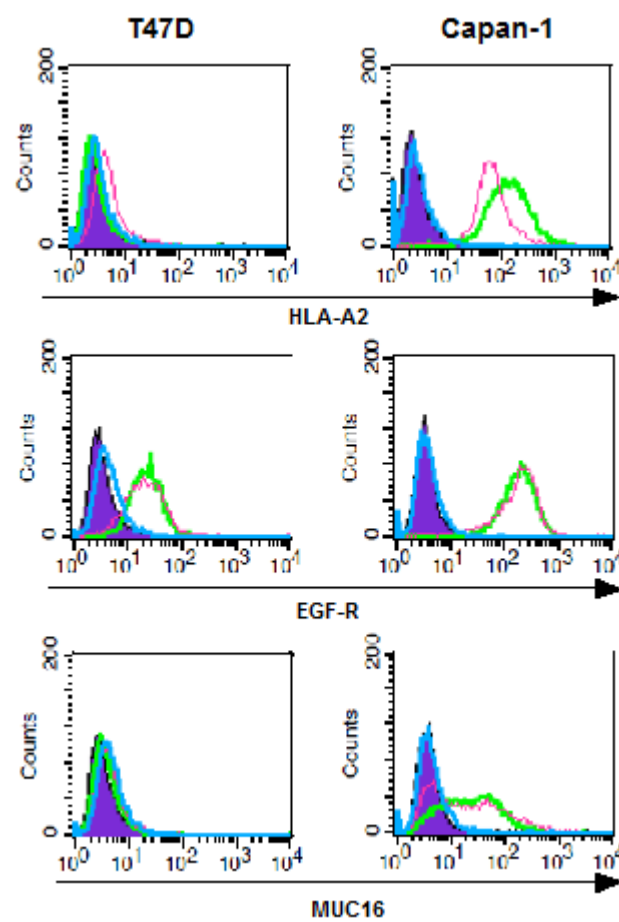

Supplement: Figure S2 — Target expression profile. Flow cytometry staining using non-perm WT (green) and COSMC KO (pink) cells to quantify the surface expression of HLA-A2, EGF-R (Erbitux®) and MUC16 (M11). Isotype control for WT (purple) and KO (blue) was used as background control. (PDF) [file pone.0072413.s002.pdf]

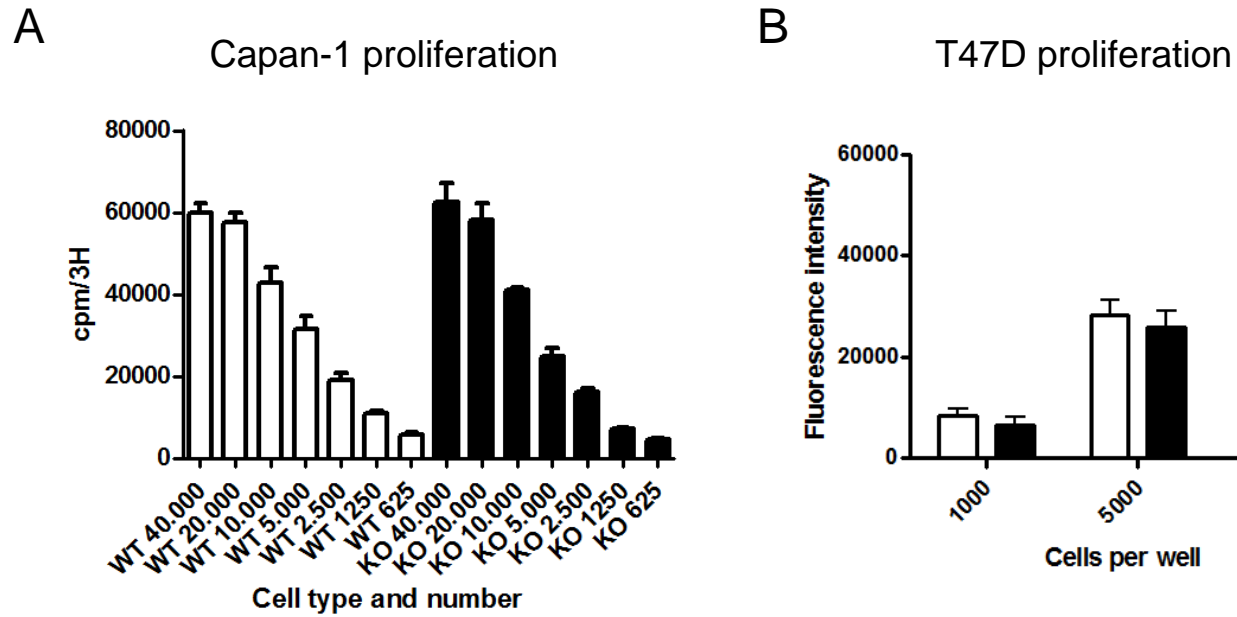

Supplement: Figure S3 — Proliferation profile for Capan-1 and T47D WT and COSMC KO cells. A) Thymidine(3H) incorporation is shown for individual cell concentrations of WT and COSMC KO Capan-1 cells after 18 H of incubation. Cpm: counts per minute. Representative of two individual experiments on two different cell passage numbers/batches. B) Fluorescence intensity in wells after CyQuant® proliferation assay of T47D WT and COSMC KO cells. Representative data set of 3 individual experiments. (PDF) [file pone.0072413.s003.pdf]

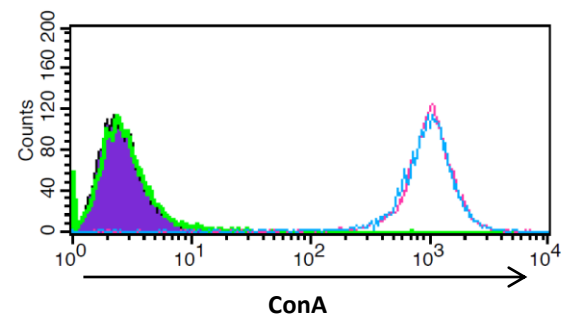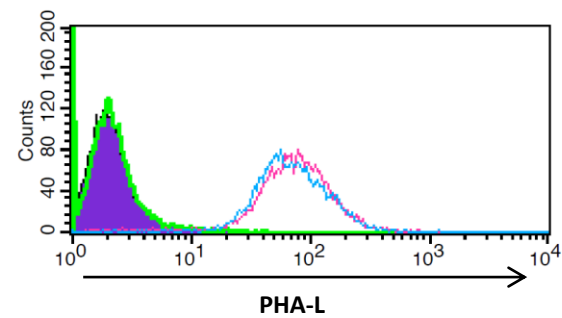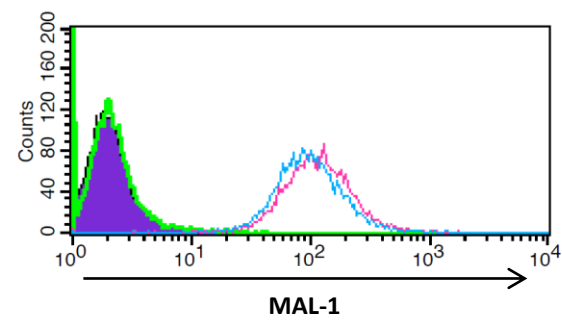

Supplement: Figure S4 — Lectin staining of WT and COSMC KO capan-1 cells. Flow cytometric staining of WT (pink) and KO (blue) cells with ConA, PHA-L and MAL-1. Non stained (purple) and streptavidin alone (green) used as background. (PDF) [file pone.0072413.s004.pdf]

## Donor A

ADCC Capan-1  
transient COSMC rescue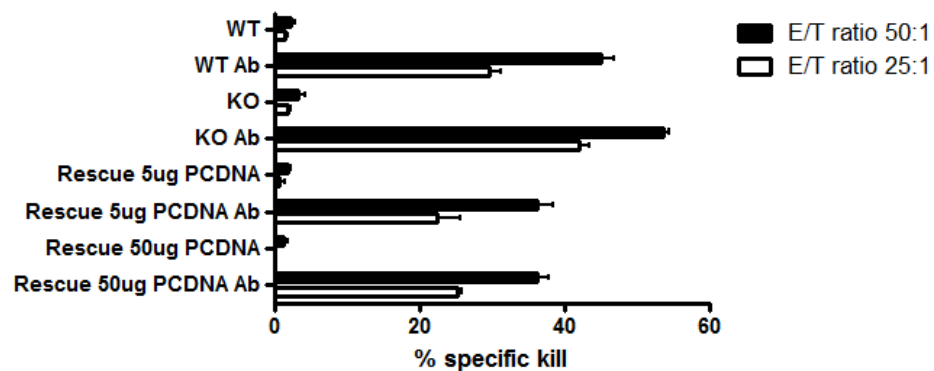

## Donor B

ADCC Capan-1  
transient COSMC rescue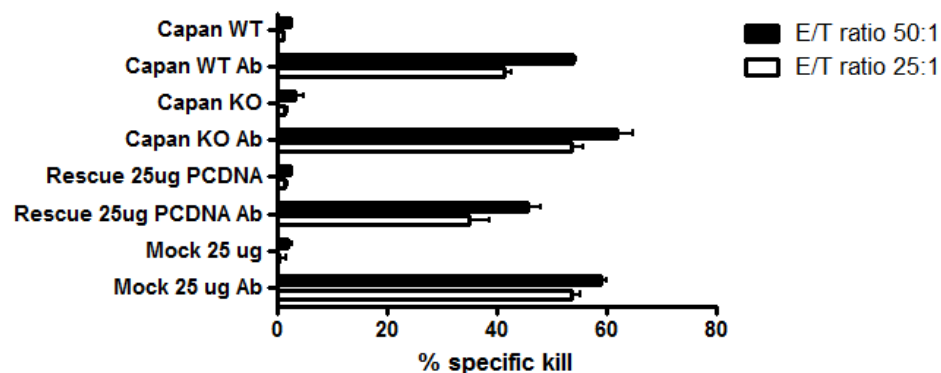

Supplement: Figure S5 — Data on additional rescue setup. Transfections performed with different batches of PCDNA3 construct and different concentrations. Analysis as in figure 2. (PDF) [file pone.0072413.s005.pdf]

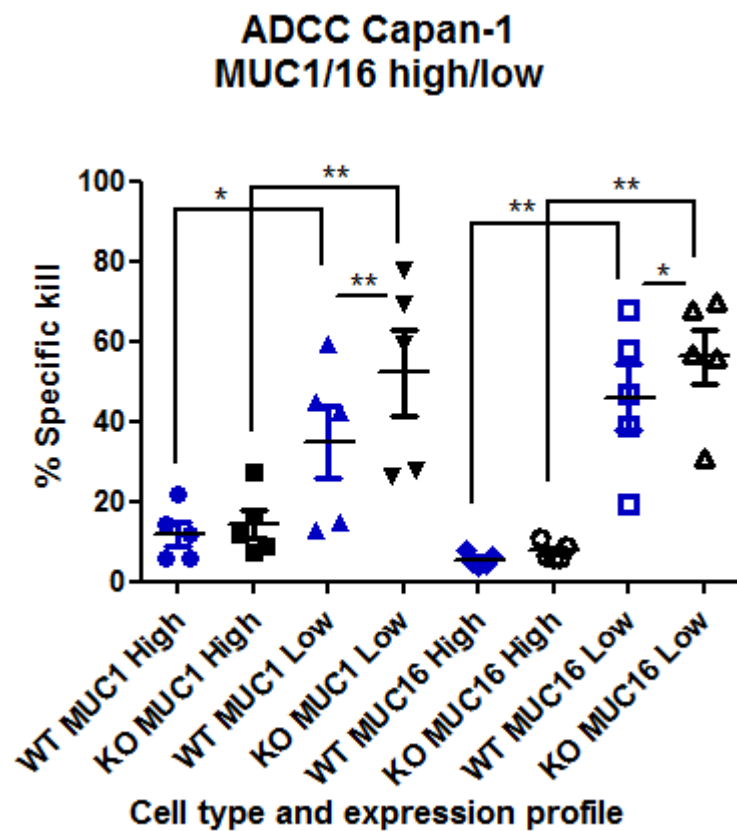

Supplement: Figure S6 — Pooled ADCC data on mucin high and low expressing cells. Equivalent % specific kill as depicted in figure 4. Paired students t-test results in significant difference with WT MUC1 High/Low: *P = 0.02, KO MUC1 High/Low: **P = 0.0082, MUC1 Low WT/KO: **P = 0.0012, WT MUC16 High/Low: **P = 0.0092, KO MUC16 High/Low: **P = 0.0024, and MUC16 Low WT/KO: *P = 0,013. (PDF) [file pone.0072413.s006.pdf]

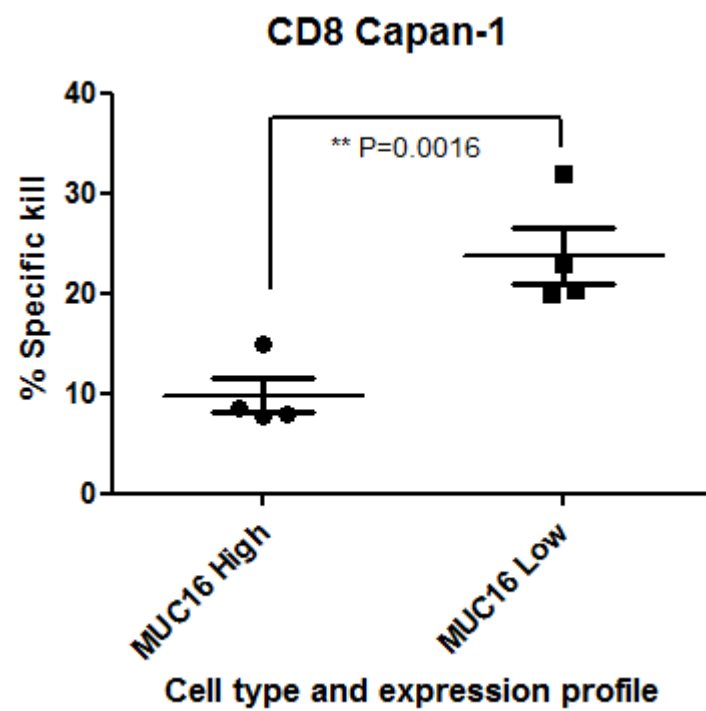

Supplement: Figure S7 — Pooled CD8+ T cell kill data on MUC16 high and low expressing cells. Equivalent % specific kill as depicted in figure 5. Paired students t-test results in significant difference with P = 0,0016. (PDF) [file pone.0072413.s007.pdf]
