# Supplementary material for: Glycan Elongation Beyond the Mucin Associated Tn Antigen Protects Tumor Cells from Immune-Mediated Killing
Source: PLoS One. 2013 Sep 6;8(9):e72413. doi: 10.1371/journal.pone.0072413 (PMC3765166; doi:10.1371/journal.pone.0072413)
Supplement: Table S1 — ADCC data for all donors, of which representative donors are shown in figure 2 . Stars indicate level of significance. P value for individual experiments obtained by unpaired students t test, while P value for cumulative data (last row) was obtained by paired students t test. § average % specific kill was slightly above 100%, set to 100% in analysis. N/A: not available, N/S: not significant. (PDF) [file pone.0072413.s008.pdf]

% specific kill E:T 50:1 for individual donors

Cell line	Modification	1	2	3					Mean
T47D	WT	9,1	13,6	13,9					12,2
	COSMC KO	12,5	18,6	19,7					16,9
	P-value	**0,0036	*0,035	*0,019					*0,0215
Cell line	Modification	4	5	6	7	8	9	10	
Capan-1	WT	13,2	45,1	10,3	21,2	42,8	28,2	15,3	25,2
	COSMC KO	20,2	53,8	17,9	24,9	50,3	37,3	25,4	32,8
	P-value	**0,002	<0,0001	<0,0001	*0,044	*0,0264	***0,0003	<0,0001	**** <0,0001
Cell line	Modification	11	12	13					
Capan-1	WT	13,3	48,9	39,4					33,9
	COSMC KO	27,6	61,5	57,2					48,8
	WT + neu	18,5	100§	72,3					63,6
	COSMC KO + neu	N/A	79,6	66,9					73,25
	P-value WT	0,0002	<0,0001	<0,0001					N/S P= 0,15
	P-value KO	N/A	***0,0007	**0,0027					N/A
